# Dyslipidemia and associated factors among adult type two diabetes mellitus patients in Felege Hiywot Refral, Hospital, Bahir Dar, Ethiopia, 2023

**DOI:** 10.3389/fcvm.2024.1493447

**Published:** 2024-12-20

**Authors:** Zemenu Addis, Ayenew Tega Nega, Robel Demelash Tebeje, Engdaw Asmare, Alemu Bezabih Tegegnie, Workineh Tamir, Tamiru Alene

**Affiliations:** ^1^ Department of Clinical Nursing, Hosanna Health Science College, Hosanna, Ethiopia; ^2^ Department of Midwifery, Hosanna Health Science College, Hosanna, Ethiopia; ^3^ Department of Family Health, Hosanna Health Science College, Hosanna, Ethiopia; ^4^ Department of Dermatovenrology, College of Medicine and Health Sciences, Injibara University, Injibara, Ethiopia; ^5^ Department of Medical Laboratory Science, College of Medicine and Health Sciences, Injibara University, Injibara, Ethiopia; ^6^ Department of Pediatrics and Child Health Nursing, College of Medicine and Child Health Nursing, Injibara University, Injibara, Ethiopia

**Keywords:** type-2 diabetes, dyslipidemia, factors, Bahir Dar, Ethiopia

## Abstract

**Background:**

Dyslipidemia is a common condition in type two diabetic patients, and it is thought to have a significant role in moderating the cardiovascular risk associated with diabetes. Data on serum lipid profiles in type 2 diabetes patients from Bahir Dar, Ethiopia is limited. This study aimed to evaluate the prevalence of dyslipidemia among adult type 2 diabetes patients and to explore potential contributing factors.

**Method and materials:**

A facility-based cross-sectional study was conducted with 354 type 2 diabetes mellitus patients from April 3 to June 4, 2023. Data were collected through the use of structured questionnaires and checklists. The data were entered into EpiData version 4.6 and analyzed using SPSS version 26. Logistic regression was employed to identify variables significantly associated with the outcomes, with a *p*-value ≤ 0.05 and a 95% confidence interval.

**Results:**

A total of 369 individuals with diabetes were approached in this study, resulting in a response rate of 96%. The overall prevalence of dyslipidemia was 61.3% (95% CI: 56.2–66.7). Of those with dyslipidemia, 11% had a single serum lipid abnormality, while 50.3% had a combined serum lipid abnormality. Significant factors associated with dyslipidemia included being over 60 years old (AOR: 2.4, 95% CI: 1.2–5.0), poor fasting blood glucose control (AOR: 2.5, 95% CI: 1.2–5.1), being overweight (AOR: 5.8, 95% CI: 3.2–11), physical inactivity (AOR: 3.4, 95% CI: 1.7–7.0), and being a past alcohol drinker (AOR: 3.1, 95% CI: 1.3–7.4).

**Conclusion:**

In the study area, a high prevalence of dyslipidemia was found among diabetic patients. Independent factors associated with dyslipidemia included older age, poor fasting blood glucose control, physical inactivity, a history of alcohol consumption, and being overweight. To address this issue, it is essential to implement preventive measures such as early detection, patient education, dietary monitoring, regular clinical visits, physical exercise, and weight management. These strategies represent the most effective approach to combating dyslipidemia.

## Introduction

Diabetes mellitus is a multifactorial condition characterized by a group of metabolic disorders that result in hyperglycemia. It arises from either partial or complete deficiency of insulin (1, 2). The risk factors for T2DM are age, being overweight or obese, and being physically inactive are risk factors (2). In 2021, it is estimated that more than 6.7 million people aged 20–79 will die from diabetes-related complications (3). The global prevalence of diabetes among individuals aged 20–79 was 10.5%, affecting 536.6 million people. This figure is projected to rise to 12.2%, impacting 783.2 million people by the year 2045 (4).

Dyslipidemia is prevalent among individuals with type 2 diabetes and is believed to play a significant role in influencing the cardiovascular risk associated with diabetes (5). Dyslipidemia, is a risk factor for vascular issues in diabetic patients, which is worsened by elevated levels of inflammatory adipokines (6–8).

Blood pressure, fasting blood glucose, body mass index (BMI), age, insufficient physical activity, dietary habits, tobacco use, and other lifestyle behaviors have been identified as the main risk factors for the rising prevalence of dyslipidemia among T2DM patients (9, 10). Individuals diagnosed with T2DM frequently exhibit increased amounts of low-density lipoproteins, raised triglycerides, and low high-density lipoproteins (11).

Estimates from the Global Burden of Disease research in 2019 indicated that elevated levels of low-density lipoprotein cholesterol (LDL-C) were responsible for 3.78 million deaths from ischemic heart disease worldwide, accounting for 44.3% of all ischemic heart disease fatalities. Additionally, high LDL-C levels contributed to 0.61 million deaths from ischemic stroke, representing 22.4% of all ischemic stroke deaths (11). The global prevalence of dyslipidemia is rising steadily, likely driven by factors such as Westernized diets, decreased physical activity, urbanization, and increasing obesity (9, 12, 13). Dyslipidemia is also highly prevalent in developing countries, including Ethiopia (9, 14–16).

There is insufficient data on the prevalence of dyslipidemia among type 2 diabetes mellitus (T2DM) patients in the study area. Identifying the factors linked to dyslipidemia is essential for crafting a targeted approach. Therefore, this study sought to investigate the prevalence of dyslipidemia and its associated factors among diabetic patients in the region.

## Methods and materials

### Study design, study area, and period

A facility-based cross-sectional study was conducted from April 3 to June 4, 2023, involving adult outpatients at the diabetes clinic of Felege Hiywot Referral Hospital in Bahir Dar, Ethiopia. This study took place in the chronic follow-up units, located within the outpatient department, which offers regular appointments for patients with chronic conditions such as hypertension, heart disease, and diabetes. Follow-up services in this unit were provided by both a nurse and a physician.

### Source population

All T2DM patients attending outpatient departments in Felege Hiywot Referal Hospital were the source population.

### Study population

T2DM patients who fulfilled the inclusion criteria during the data collection period were the study population.

### Sample size and sampling technique

The required sample size was determined using a single population proportion formula with the assumption of a confidence interval of 95%, a margin of error of 5% $\left(n=\frac{\left(z_{\frac{\alpha }{2}}\right)2*p(1-P)}{d^{2}}\right)$, considering 68.1% of the overall proportion of dyslipidemia among adult patients with T2DM a study conducted at Jimma University Medical Center, Jimma, Southwest Ethiopia (9). When we substitute $\frac{{\left(1.96\right)}^{2}*0.68.1*0.319}{{\left(0.05\right)}^{2}}=333.8$.

By considering adjustment for the expected non-response rate of 10% the total sample size became 369.

A non probability convenience sampling procedure was utilized to collect required data from strudy unit. because it would have been challenging to deploy other probability sampling techniques because of the varieties within the potential study members, and follow-up arrangement, a few of them may not have returned to the clinic on the required date as result it was difficult to access them and even more participants were not volunteer to provide data.

### Exclusion and inclusion criteria

Type 2 diabetes mellitus patients aged 18 years and older who visited the outpatient diabetes clinic during the data collection period and had at least three month of follow-up outpatient care, were included in the study. Exclusion criteria included pregnancy, incomplete medical recored, and critical illness.

### Operational definition

Dyslipidemia was identified in individuals with abnormalities in their lipid profile, whether singular or combined. It was characterized by one or more of the following lipid profile anomalies in T2DM patients: total cholesterol (TC) ≥ 200 milligrams per deciliter (mg/dl), low-density lipoprotein (LDL) ≥ 100 mg/dl, triglycerides (TG) ≥ 150 mg/dl, or high-density lipoprotein (HDL) ≤ 40 mg/dl (14). Glycemic control was classified as either poor or good. Good glycemic control was defined as an average fasting blood glucose (FBG) level of 80–130 mg/dl (4.4–7.2 mmol/L) and poor glycemic control was indicated by an FBG level greater than 130 mg/dl (>7.2 mmol/L) over three months (17). Social support was categorized with the Oslo 3 social support scale and participants who scored 3–8, 9–11, and 12–14 out of 14 were considered as having poor, moderate, and strong social support respectively (18). Physical Activity: The Short-Form International Physical Activity Questionnaire (IPAQ) was used to evaluate participants' physical activity levels in this study. The IPAQ is known for its good reliability and validity, with a standard Cronbach's *α* coefficient of 0.8. For diabetic individuals to be considered physically active, they needed to meet one of the following criteria: Engage in vigorous physical activity for at least 3 days a week and accumulate a minimum of 1,500 metabolic equivalent of task (MET) minutes per week or accumulate at least 3,000 MET minutes per week through any combination of walking, moderate-intensity, or vigorous-intensity activities over 7 or more days or engage in moderate-intensity activity and/or walking for at least 30 min per day on 5 or more days a week. Those who did not meet any of these criteria were classified as physically inactive (19, 20).

### Study variables

The dependent variable in this study was dyslipidemia among people with T2DM. Independent variables are socio-demographic variables (age, sex, educational level, occupation, income, health care cost, and residence), individual lifestyle and psychosocial-related variables (smoking, alcohol drinking, dietary adherence, physical activity, social support, and depression), and clinical variables (type of treatment modality, family history of T2DM, DM related complications, DM duration, body mass index, hypertension, and glycemic control).

### Data collection tools

Structured questionnaires were used to collect information on the risk variables that could be connected to dyslipidemia. The questionnaire contains respondent socio-demographic characteristics, lifestyle and psychosocial characteristics, and clinical characteristics. Data extraction checklists were developed based on reviewed works of literature (6, 9, 14, 16).

Participants were asked to submit sociodemographic data (age, sex, educational level, occupation, income, and residence), individual lifestyle, and psychosocial-related variables (smoking, alcohol drinking, dietary adherence, physical activity, social support, and depression). Data on the study participants' including total cholesterol (TC), triglycerides (TG), high-density lipoprotein cholesterol (HDL-C), low-density lipoprotein cholesterol (LDL-C), and fasting blood glucose (FBG) levels were gathered from their medical records if they had been measured during the three months leading up to the research, provided that the individual's medication had not changed over that period. If this was not the case, then they were tested during the research.

### Lifestyle and psychosocial characteristics

#### Smoking status

Based on the history of smoking status, the WHO STEPS questionnaire classifies patients as current smokers if they had smoked in the 30 days preceding the data collection period, former smokers if they had smoked at least 100 cigarettes in their lifetime but had quit smoking by the time of the interview, and never smokers if they had never smoked or had smoked fewer than 100 cigarettes in their lifetime (21).

#### Alcohol drinking habit

Based on the history of alcohol consumption, the WHO STEPS questionnaire classifies patients as current alcohol drinkers if they consumed alcohol within the 30 days before data collection. Patients are categorized as former alcohol drinkers if they had consumed alcohol at any point in the last 12 months but did not do so in the past 30 days (22).

#### Depression

On PHQ-9 depression subscales patients were classified as having minimal depression if the PHQ-9 score is 0–4, mild depression if the PHQ-9 score is 5–9, moderate depression if the PHQ-9 score is 10–14, moderately severe depression if the PHQ-9 score is 15 –19, and severe depression if the PHQ-9 score is 20–27 (23).

#### Adherence to dietary regimen

On the perceived dietary adherence questionnaire (PDAQ), patients were classified as adhered if they scored at least four days in the week on the dietary adherence questionnaire and non-adherent if they scored less than four days in the week (24).

### Clinically related variables

#### Body mass index (BMI)

Each study participant's height and weight were measured according to World Health Organization (WHO) guidelines. Body mass index (BMI) was then calculated by dividing weight in kilograms by the square of height in meters. The BMI results were categorized as follows: underweight if the BMI was less than 18.5 kg/m^2^, healthy weight if the BMI ranged from 18.5 to 24.9 kg/m^2^, overweight if the BMI was between 25 and 29.9 kg/m^2^, and obese if the BMI was 30 kg/m^2^ or higher (20).

#### Blood pressure measurement

Blood weight was carefully measured using a Small Scale Life BP A50 (Miniaturized Scale Life AG, Switzerland) in accordance with World Health Organization guidelines. Blood pressure was recorded with a mercury sphygmomanometer from the participant's upper arm after they had been seated quietly for 5 min. Hypertension was defined as a systolic blood pressure (SBP) greater than 140 millimeters of mercury (mmHg) or a diastolic blood pressure (DBP) greater than 90 mmHg in diabetic patients.

### Data quality control

Data were collected using pre-tested structured questionnaires from 5% of the participants at Injibara General Hospital. Based on the pre-test results, minor adjustments were made to the questionnaires, including editing any terms that were not easily understood by the respondents. Four nurses with bachelor's degrees were recruited for data collection, and two MSc students were appointed as supervisors.

### Data analysis

The data were first entered into Epi-data version 4.6 and then exported to SPSS version 26 for analysis. Descriptive statistics were employed to summarize the variables in terms of frequency, mean, and percentages. Cross-tabulation was performed for all variables. Variables with a *p*-value ≤ 0.2 in the bivariable analysis were included in the multivariable logistic regression. In the multivariable regression, a *p*-value ≤ 0.05 with a 95% confidence interval was deemed statistically significant. The adjusted odds ratios (AOR) and their corresponding 95% confidence intervals were reported as measures of association.

## Results

### Sociodemographic characteristics of the study participants

A total of 369 individuals with diabetes were approached in this study, resulting in a response rate of 96%. Among the respondents, 63% of female participants were found to have dyslipidemia. The mean age of the participants was 51.71 years. Additionally, 65.3% of the rural residents in the study had dyslipidemia (Table 1).

**Table 1:** Sociodemographic characteristics of adult T2DM patients in Felge Hiyowt Hospital, Bahiradr Ethiopia, 2023.

Variables	Category	Dyslipidemia	
		Yes	No
Sex	Male	108 (59.7%)	73 (40.3%)
	Female	109 (63.0%)	64 (37.0%)
Age	>60 years	122 (76.3%)	38 (23.8%)
	40–60 years	52 (50.5%)	51 (49.5%)
	<40 years	43 (47.3%)	48 (52.7%)
Residence	Urban	121 (58.5%)	86 (41.5%)
	Rural	96 (65.3%)	51 (34.7%)
Education status	Unable to read and write	41 (60.3%)	27 (39.7%)
	Read and write only	38 (56.7%)	29 (43.3%)
	Primary school	26 (68.4%)	12 (31.6%)
	Secondary school	26 (57.8%)	19 (42.2%)
	College and above	86 (63.2%)	50 (36.8%)
Occupation	Government employee	50 (59.5%)	34 (40.5%)
	Retired	20 (60.6%)	13 (39.4%)
	Housewife	37 (57.8%)	27 (42.2%)
	Merchant	36 (60.0%)	24 (40.0%)
	Farmer	45 (64.3%)	25 (35.7%)
	Private employee	29 (67.4%)	14 (32.6%)
Health care cost coverage	Self	106 (60.2%)	70 (39.8%)
	Employ organization	16 (61.5%)	10 (38.5%)
	Health insurance	90 (62.1%)	55 (37.9%)
	NGO	5 (71.4%)	2 (28.6%)
Average monthly income	Above 2,500 birr	83 (59.7%)	56 (40.3%)
	1,000–2,500 birr	86 (61.0%)	55 (39.0%)
	Less than 1,000 birr	48(64.9%)	26(35.1%)

### Lifestyle and psychosocial characteristics of the study participants

Among the participants in this study, 79% of those who were physically inactive had dyslipidemia. The prevalence of dyslipidemia was highest in groups with poor social support (69.2%), mild depression (66.9%), and those with a history of alcohol consumption (81.3%) (Table 2).

**Table 2:** Lifestyle and psychosocial characteristics of adult T2DM patients in Felge Hiyowt Hospital, Bahiradr Ethiopia, 2023.

Variables	Categories	Dyslipidemia%	
		Yes	No
Social support	Poor	92 (69.2%)	41 (30.8%)
	Moderate	65 (56.0%)	51 (44.0%)
	Strong	60 (57.1%)	45 (42.9%)
Physical activity	Low	132 (79.0%)	35 (21.0%)
	Moderate	40 (40.0%)	60 (60.0%)
	High	45 (51.7%)	42 (48.3%)
Alcohol drinking	Past drinker	113 (81.3%)	26 (18.7%)
	Current drinker	35 (57.4%)	26 (42.6%)
	Non drinker	69 (44.8%)	85 (55.2%)
Dietary adherence	Non adhered	84 (55.3%)	68 (44.7%)
	Adhered	13 (365.8%)	69 (34.2%)
Smoking	Past smoker	89 (75.4%)	29 (24.6%)
	Current smoker	50 (55.6%)	40 (44.4%)
	Non-smoker	78 (53.4%)	68 (46.6%)
Levelof depression	Moderate depression	19 (61.3%)	12 (38.7%)
	Mild depression	107 (66.9%)	53 (33.1%)
	No depression	91(55.8%)	72(44.2%)

### Clinical characteristics of adult T2DM patients

The mean duration of diabetes was 7.46 years. Oral hypoglycemic (63.5%) was a commonly utilized therapy. The prevalence of dyslipidemia was highest among those who were overweight (82.2%) as compared to healthy weight. Of the study participants who had diabetes-related complications (67.2%) had abnormal serum lipid profiles (Table 3).

**Table 3:** Clinical characteristics of adult T2DM patients in Felge Hiyowt Hospital, Bahiradr Ethiopia, 2023.

Variables	Categories	Dyslipidemia%	
		Yes	No
Type of treatment modality	Insulin and oral	86 (66.7%)	43 (33.3%)
	Oral only	131 (58.2%)	94 (41.8%)
Durationof diabetes	>10 years	83 (57.6%)	61 (42.4%)
	5–10 years	59 (60.8%)	38 (39.2%)
	<5 years	75 (66.4%)	38 (33.6%)
Family history of DM	No	154 (59.5%)	105 (40.5%)
	Yes	63 (66.3%)	32 (33.7%)
Fasting blood glucose	Poor control	130 (52.0%)	120 (48.0%)
	Good control	87 (83.7%)	17 (16.3%)
Hypertension	No	107 (58.2%)	77 (41.8%)
	Yes	110 (64.7%)	60 (35.3%)
DM complication	No	90 (54.5%)	75 (45.5%)
	Yes	127 (67.2%)	62 (32.8%)
Body mass index (BMI	Obese	18 (48.6%)	19 (51.4%)
	Overweight	129 (82.2%)	28 (17.8%)
	healthy weight	70(43.8%)	90(56.3%)

### Pattern of serum lipid profile abnormality among adult T2DM patients (*n* = 354)

The overall prevalence of dyslipidemia in the study was 61.3% (95% CI: 56.2–66.7). Our findings indicated that 11% of the study population had a single lipid abnormality, while 50.3% had combined serum lipid abnormalities. Among those with a single abnormality, low LDL-C was present in 52.2% of the population. In contrast, combined lipid profile abnormalities, including elevated TG, TC, LHDL-C, and LDL-C, were observed in 16.9% of the study population (Table 4).

**Table 4:** Pattern of serum lipid profile abnormality among adult T2DM patients (*n* = 354).

		Frequency	Percentage
	Dyslipidemia	217	61.3
Single serum lipid abnormality	HDL-C < 40 mg/dl	19	5.3
	LDL-C ≥ 100 mg/dl	12	3.3
	TG ≥ 150 mg/dl	5	1.4
	TC ≥ 200 mg/dl	3	0.84
Combined plus isolated	LDL	185	52.2
	HDL	117	33.0
	TG	152	42.9
	TC	148	41.8

HDL-C, high-density lipoprotein cholesterol; LDL-C, low-density lipoprotein cholesterol; TC, total cholesterol; TG, triglyceride.

### Factors associated with dyslipidemia among adult T2DM patients

In bivariate analysis: age, residency, dietary adherence, smoking, alcohol drinking, hypertension, physical activity, fasting blood glucose, depression, BMI, and DM complications were significantly associated with dyslipidemia. After adjusting for possible confounding factors respondent's age, level of physical activity, alcohol drinking, fasting blood glucose control, and BMI were significantly associated with a *p*-value of 0.05 at 95% CI.

Individuals aged over 60 years had twice the odds of developing dyslipidemia compared to those under 40 years (AOR: 2.4, 95% CI: 1.2–5.0). Diabetic individuals with poor fasting blood glucose control were also more than twice as likely to have dyslipidemia compared to those with good control (AOR: 2.5, 95% CI: 1.2–5.1). Overweight individuals had more than five times the likelihood of developing dyslipidemia compared to those with a healthy weight (AOR: 5.8, 95% CI: 3.2–11.0). The odds of dyslipidemia were over three times higher in physically inactive individuals compared to those who were active (AOR: 3.4, 95% CI: 1.7–7.0). Additionally, past drinkers had three times the risk of developing dyslipidemia compared to non-drinkers (AOR: 3.1, 95% CI: 1.3–7.4) (Table 5).

**Table 5:** Bivariable and multivariable logistic regression analysis results of factors associated with dyslipidemia among adult T2DM patients (*n* = 354), 2023.

Variables	Category	Dyslipidemia		COR	AOR(95%CI)	*P* value
		Yes	No			
Residence	Urban	43 (47.3%)	1	1		
	Rural	121 (58.5%)	86 (41.5%)	1.33		
Age	>60 years	122 (76.3%)	38 (23.8%)	3.6	2.4 (1.2–5.0)	0.015
	40–60 years	52 (50.5%)	51 (49.5%)	1.1	(0.5–1.9)	
	<40 years	43 (47.3%)	48 (52.7%)	1	1	
Smoking status	Past smoking	89 (75.4%)	29 (24.6%)	2.7		
	Current smoking	50 (55.6%)	40 (44.4%)	1.0		
	Nonsmoker	78 (53.4%)	68 (46.6%)	1	1	
Dietary adherence	Non adhered	84 (55.3%)	68 (44.7%)	1.6		
	Adhered	13 (16.25%)	69 (86.3%)	1		
Alcohol drinking	Past drinker	113 (81.3%)	26 (18.7%)	5.4	3.1 (1.3–7.4)	0.011
	Current drinker	35 (57.4%)	26 (42.6%)	1.7	(0.9–5.3)	
	Non drinker	69 (44.8%)	85 (55.2%)	1	1	
Level of physical activity	Inactive	132 (79.0%)	35 (21.0%)	3.5	3.4 (1.7–7.0)	0.001
	Moderate intensity	40 (40.0%)	60 (60.0%)	0.6	(0.2–1.3)	
	Vigorous intensity	45 (51.7%)	42 (48.3%)	1	1	
Fasting blood glucose	Poor control	130 (52.0%)	120 (48.0%)	4.7	2.5 (1.2–5.1)	0.009
	Good control	87 (83.7%)	17 (16.3%)	1	1	
Depression	Moderate depression	19 (61.3%)	12 (38.7%)	1.3		
	Mild depression	107 (66.9%)	53 (33.1%)	1.6		
	No depression	91 (55.8%)	72 (44.2%)	1	1	
BMI	Obese	18 (48.6%)	19 (51.4%)	1.5	(0.2–1.7)	
	Overweight	129 (82.2%)	28 (17.8%)	6.0	5.8 (3.2–11)	0.000
	Healthy weight	70 (43.8%)	90 (56.3%)	1	1	
Hypertension	No	107 (58.2%)	77 (41.8%)	1	1	
	Yes	110 (64.7%)	60 (35.3%)	1.3		
DM complication	No	90 (54.5%)	75 (45.5%)	1	1	
	Yes	127 (67.2%)	62 (32.8%)	1.7		

DM, diabetes mellitus, BMI, body mass index, AOR, adjusted odds ratio, COR, crude odds ratio, CI, confidence interval, *p < 0.05, **p < 0.00.

## Discussion

In this study, the overall prevalence of dyslipidemia was 61.3%, which is nearly comparable to the rate reported in a study from Kembata Tembaro, Ethiopia (65.5%) (14), and Jimma, Ethiopia (63.5%) (9). The observed prevalence of dyslipidemia may be partly attributed to the current trend toward urbanization and the high prevalence of risky lifestyle behaviors, which increase susceptibility to serum lipid abnormalities. Our study found alarming rates of abnormal serum lipid profiles, with low LDL, low HDL, high TG, and high TC levels at 52.2%, 33%, 42.9%, and 41.8%, respectively. Previous studies in Southern Kembata Tembaro and Jimma, Ethiopia, also reported high prevalence rates for LDL, TG, HDL, and TC. Dyslipidemia was most common among individuals over 60 years old (76.3%), those who were overweight (82.2%), physically inactive (79.0%), and former alcohol drinkers (81.3%). Therefore, routine screening, monitoring for lipid abnormalities, effective weight management strategies, promoting regular physical activity, and health education become crucial.

The result of our current finding is less than the finding reported from Tanzania (83%) (25), Thailand (88.9%) (6), and Nepal (88.1%) (26). The variation in dyslipidemia prevalence across studies may be due to differences in dyslipidemia cut-off values, potential genetic predispositions, and variations in patient characteristics. The finding of our study is much higher when compared to the study conducted in China (34.64%) (27). These differences may be due to variations in awareness levels, country development, treatment protocols, follow-up practices, and glycemic control strategies between countries. Factors such as limited awareness of the complications associated with high blood sugar and poor adherence to dietary and medication recommendations could also contribute.

Our study found a statistically significant association between dyslipidemia and poor fasting blood sugar control. This result was consistent with a study conducted in Jimma, Ethiopia (9). The possible reason might be a diet with a high glycemic index was associated with an increased risk of developing dyslipidemia. Lipid and glucose metabolism are interdependent in numerous ways. Diabetic dyslipidemia is characterized by higher TC, low high-HDL-C, and LDL particles (5). Encouraging the role of lifestyle changes, such as diet and exercise, in managing both dyslipidemia and blood sugar levels. Patients should be informed about the importance of managing both conditions to reduce their risk of cardiovascular diseases and other complications.

The result also showed that increased age was positively associated with dyslipidemia. This finding was consistent with studies conducted in India (28) and Turkish (29). Though no evidence has yet been identified that age is directly associated with lipid abnormality, poor physical activity, insulin resistance, and degenerative processes might be associated with age (28, 29). Given the higher risk of dyslipidemia with advancing age, healthcare providers should prioritize regular lipid screening for older adults. Early detection can facilitate timely management and reduce the risk of cardiovascular diseases.

In this study, dyslipidemia was significantly associated with overweight. Study participants who were overweight were more likely to have dyslipidemia compared to healthy weight. A similar observation was reported from Jimma, Ethiopia (9), Kenya (15) and China (27). Overweight causes lipolysis, which releases high levels of free fatty acids into the bloodstream. This, in turn, leads to hypertriglyceridemia, as fat and muscle tissues are deficient in lipoprotein lipase. Consequently, the liver produces increased amounts of LDL and triglycerides (30, 31). Treatment plans for dyslipidemia should incorporate interventions aimed at addressing overweight. This might include personalized weight loss programs, nutritional counseling, and exercise recommendations, in conjunction with lipid-lowering medications if necessary.

The finding revealed that a T2DM patient with past alcohol drinking was associated with developing dyslipidemia when compared to non-drinkers. This finding was consistent with other studies from Ethiopia (9, 14), China (27), Bangladesh (16) and Saudi Arabia (12). Alcohol can disrupt various lipid levels in the body and has been associated with elevated triglycerides (32). For T2DM patients with a history of alcohol use, treatment plans for dyslipidemia should address this risk factor. This might include counseling on the long-term effects of alcohol on lipid levels and integrating strategies to manage any residual impact of past alcohol use.

## Limitation of the Study

Due to budget constraints, some important variables, such as HbA1c levels, liver enzymes, and renal function tests, were not included. Additionally, the study did not assess the lifetime burden of risk factors.

## Conclusion

The study revealed a high prevalence of dyslipidemia among patients with type 2 diabetes mellitus attending the outpatient department in the study area. Independent factors associated with dyslipidemia included age, alcohol consumption, BMI, fasting blood sugar levels, and insufficient physical activity. To address this issue, it is essential to implement preventive measures such as early detection, patient education, dietary monitoring, regular clinical visits, physical exercise, and weight management. These strategies represent the most effective approach to combating dyslipidemia.

## Data Availability

The original contributions presented in the study are included in the article/Supplementary Material, further inquiries can be directed to the corresponding author.
